# Cannabidiol as a treatment for arthritis and joint pain: an exploratory cross-sectional study

**DOI:** 10.1186/s42238-022-00154-9

**Published:** 2022-08-24

**Authors:** Nicholas Frane, Erik Stapleton, Cesar Iturriaga, Maximillian Ganz, Vijay Rasquinha, Robert Duarte

**Affiliations:** 1 The Core Institute/Banner University Medical Center – Phoenix, 18444 N 25th Ave #210, Phoenix, AZ USA; 2 Department of Orthopaedic Surgery, New England Baptist Hospital, 125 Parker Hill Ave, Boston, MA USA; 3 Department of Orthopaedic Surgery, Zucker School of Medicine at Hofstra/Northwell, 270 Park Ave, Huntington, NY USA; 4 Department of Orthopaedic Surgery, Zucker School of Medicine at Hofstra/Northwell, Long Island Jewish Hospital, Northwell Health, Great Neck, NY USA; 5 Zucker School of Medicine at Hofstra/Northwell, Pain Institute of Neurology, Long Island Jewish Hospital, Northwell Health, Great Neck, NY USA

**Keywords:** Cannabidiol, CBD research, CBD arthritis, CBD for pain, Cannabis medicine, Orthopedic surgery, Arthritis treatment

## Abstract

**Introduction:**

An estimated 54 million Americans currently suffer from debilitating arthritis. Patients who have exhausted conservative measures can be subject to chronic pain and resort to symptomatic management with anti-inflammatories, acetaminophen, and opioids. Cannabidiol (CBD) is a non-psychoactive cannabinoid that has shown promise in preclinical studies to reduce inflammation and pain associated with arthritis. The purpose of this study was to explore patient perceived effects of cannabidiol on symptoms of arthritis.

**Methods:**

A novel anonymous questionnaire was created to evaluate perceived efficacy of cannabidiol for the treatment of arthritis. A self-selected convenience sample (*N*=428) was recruited through online methods including social media accounts and newsletters (The Arthritis Foundation and Savvy Cooperative) between May 5, 2020, and November 5, 2020. Statistical analysis was performed to determine differences between types of arthritis and improvements in quality-of-life symptoms. Furthermore, a regression analysis was performed to identify variables associated with decreasing or discontinuing other medications.

**Results:**

CBD use was associated with improvements in pain (83%), physical function (66%), and sleep quality (66%). Subgroup analysis by diagnosis type (osteoarthritis, rheumatoid, or other autoimmune arthritis) found improvements among groups for physical function (*P*=0.013), favoring the osteoarthritis group. The overall cohort reported a 44% reduction in pain after CBD use (*P*<0.001). The osteoarthritis group had a greater percentage reduction (*P*=0.020) and point reduction (*P*<0.001) in pain compared to rheumatoid arthritis and other autoimmune arthritis. The majority of respondents reported a reduction or cessation of other medications after CBD use (*N*=259, 60.5%): reductions in anti-inflammatories (*N*=129, 31.1%), acetaminophen (*N*=78, 18.2%), opioids (*N*=36, 8.6%) and discontinuation of anti-inflammatories (*N*=76, 17.8%), acetaminophen (*N*=76, 17.8%), and opioids (*N*=81, 18.9%).

**Conclusion:**

Clinicians and patients should be aware of the various alternative therapeutic options available to treat their symptoms of arthritis, especially in light of the increased accessibility to cannabidiol products. The present study found associations between CBD use and improvements in patient’s arthritis symptoms and reductions in other medications. Future research should focus on exploring the benefits of CBD use in this patient population with clinical trials.

**Supplementary Information:**

The online version contains supplementary material available at 10.1186/s42238-022-00154-9.

## Introduction

Cannabidiol (CBD) and delta-9-tetrahydrocannabinol (THC) are the most prevalent, pharmacologically active phytocannabinoids found in the *Cannabis sativa* plant. THC has well-established effects on pain but is associated with psychoactive effects, which may hinder its application in the pain-reduction setting (McCarberg, 2007). In contrast, CBD lacks the psychoactive properties of THC but has anti-inflammatory, antinociceptive, and antioxidant effects, making it an intriguing target for the treatment of pain and inflammatory conditions, including the numerous manifestations of arthritis (Atalay et al., 2020; Iffland & Grotenhermen, 2017). Previous studies have identified the potential for cannabinoids to directly modulate pain within synovial joints (Richardson et al., 2008; N. Schuelert et al., 2010; Niklas Schuelert & McDougall, 2008). Specifically, basic science studies have depicted that the endocannabinoid system (ECS) is prevalent within the synovium and can regulate inflammation and nociception in both humans and animal synovial joints (Hammell et al., 2016; Malfait et al., 2000; Richardson et al., 2008). This growing body of literature is beginning to indicate that cannabinoids target the ECS within arthritic joints and may confer mediation to arthritic and inflammatory joint disease (Richardson et al., 2008). Furthermore, preclinical studies have shown that CBD reduces inflammation and pain behaviors in animal models with arthritis (Hammell et al., 2016; Whyte et al., 2012). While not regulated by the U.S. Food and Drug Administration (FDA), positive public perception has led to an increase in the utilization of CBD as a treatment option for various medical conditions. Recent literature indicates that 62% of CBD users are treating a medical condition, of which anxiety, chronic pain, and arthritis were the most frequent (Corroon & Kight, 2018). Few studies have looked specifically at arthritis to determine the subjective benefits of CBD use.

An estimated 23% of all adults, or 54 million Americans, are suffering from arthritis, with osteoarthritis and rheumatoid arthritis (RA) constituting the majority (*Arthritis | CDC*, n.d.). In addition, 24 million of these patients have limited activity as a result of their arthritis (*Arthritis | CDC*, n.d.). Inflammatory pathology, as a contributor to degenerative changes in the arthritic joint, is thought to lead to the development of peripheral sensitization and nociceptive pain (Krustev et al., 2015; Niklas Schuelert & McDougall, 2008, 2009). The etiologies of arthritis include chronic synovial joint disease, degenerative changes, localized inflammatory states, and autoimmune responses. Regardless of the cause and disease pathogenesis, patients with arthritis are subject to chronic pain, joint degeneration, and articular neuropathy, for which there are few viable long-term treatment options without significant side effects. Nonsteroidal anti-inflammatory drugs (NSAIDs) are commonly the first line of analgesia. However, with long-term use, the efficacy of NSAIDs decline, and they can lead to major adverse outcomes like bleeding events, peptic ulcer disease, cardiovascular events, and acute kidney injury (Lanas & Chan, 2017; Nissen et al., 2016). Furthermore, patients with progressive disease may eventually utilize opioid analgesics, which can result in dependency and addiction (Trouvin et al., 2019). Rheumatic diseases, defined by their generalized inflammatory response, represent another major source of chronic inflammatory joint pain. The advent of disease-modifying anti-rheumatic drugs has been pivotal in the treatment of RA, yet patients may still be hindered by debilitating joint pain (Ishida et al., 2018). Control of chronic joint pain remains a difficult feat, and patients commonly resort to surgical options such as total joint arthroplasty once nonoperative modalities have failed (Breivik et al., 2006; Geeske Peeters et al., 2017; Peter et al., 2015).

Given the evidence supporting CBD as a sustainable option for pain management, we believe it may be a potential alternative in treating patients with arthritis. Despite the loosened regulatory status of CBD and its increased availability to the public, research exploring patient-perceived benefits from its use for arthritic conditions is limited. The purpose of this study was to address this gap in the literature by evaluating the patient-perceived effect that CBD use has on arthritis symptoms. We sought to answer three questions: (I) Do patients with arthritis report improving or worsening symptoms with CBD use? (II) Was CBD use associated with decreases in other pharmacologic treatments for patients with arthritis? (III) Were there any differences in reported symptoms based on the type of arthritis? With this study, we aim to demystify preclinical data that suggest CBD may have a role in the treatment of arthritic conditions.

## Methods

A survey was created to evaluate participants' perceived efficacy of CBD for their arthritis symptoms. The study was granted Institutional Review Board approval at the Feinstein Institute for Medical Research, part of Northwell Health, and patient consent was not necessary because it was anonymous. The questionnaire was designed based on the outline of survey creation described by Passmore et al. (2002). Additionally, previously published literature using surveys for CBD research were referenced (Corroon et al. 2018, Bhamra et al. 2021, Moltke et al, Schilling et al. 2021, Moeller-Bertram et al. 2019, Devitt 2019).

### Survey questions

Background questions included demographic information, characteristics of CBD use, and previous treatment modalities. Questions consisted of “yes/no” and “select one of the following.” Questions regarding previous treatment modalities, the anatomical joint involved, and medications were in “check all that apply” formatting. Efficacy of CBD use for their pain and arthritis symptoms was assessed by a number of different outcome variables. Improvements in pain intensity were evaluated using a 5-point Likert scoring system, adopted from the Patient Global Impression of Change (PGIC), ranging from “much worse” to “much better.” A“Much Better” response has previously been validated and utilized as an indicator of clinically important benefit (Buchbinder et al., 1995; Farrar et al., 2001; Juniper et al., 1994; Likert, R., 1932; Salaffi et al., 2004). Furthermore, we adopted the PGIC to assess the perceived effect of CBD use on participants' sleep quality and physical function. Additionally, participants were asked to rate their arthritis pain on a 0-to-10 Numerical Rating Scale (NRS) before and after taking CBD (Cook et al., 2013). Using both a point and percentage scale, the level of pain reduction was calculated based on the difference to baseline. This question format, utilizing the NRS scale, was chosen because it has been validated as an assessment of a patient’s pain improvement, with 2.0 point reduction or 30% improvement in pain from baseline serving as a clinically important change (Farrar et al., 2001, Salaffi et al. 2004)). Lastly, participants were asked questions about reductions and cessations of medications related to their CBD use. A full list of survey questions can be seen in Appendix 1.

After survey development, multiple internal reviews by the authors were undertaken to reduce errors and to improve the accuracy of the instrument. After internal review by the authors, the survey instrument was then reviewed by the Feinstein Institute for Medical Research Clinical survey department. After a rigorous editorial process, the survey was finalized and made available for distribution.

Participants were a self-selected convenience sample who accessed the online survey between May 5, 2020, and November 5, 2020. The survey interface was facilitated through REDCap electronic data capture software (Harris et al., 2009), and a survey link was created that was subsequently distributed through various means. This included online personal and health system social media accounts as well as both the Arthritis Foundation ® and Savvy Cooperative ® groups. These aforementioned groups are patient advocacy organizations that use online social media accounts, forums, and newsletters to help provide general medical information to patients as well as establish a social network for patients to connect to one another. The flier that was utilized to recruit participants can be seen in Appendix 2. The survey data was securely collected and stored using REDCap for later statistical analysis (Harris et al., 2009). Inclusion criteria included participants ≥18 years old and those with a prior diagnosis of arthritis as a cause of joint pain. Initially, 709 respondents participated in the online survey link. Participants who answered one of the screening questions indicating they did not have arthritis (*N*=75) were excluded. Furthermore, surveys were deemed incomplete by the authors if they were missing responses regarding the patient’s diagnosis of arthritis and/or CBD use (*N*=30). Those who had not tried CBD for their arthritis pain (*N*=176) were not included in the formal analysis; however, we did compare the difference in demographics between those that used CBD and those that did not (*N*=604). Our final outcome measures were evaluated for participants who had tried CBD (*N*=428).

Participants were stratified by type of arthritis and compared for the primary outcomes, including NRS Pain improvement, PGIC for pain intensity, physical function, and sleep quality, and evaluating if CBD use reduced the use of other medications. Psoriatic arthritis, Lupus arthritis, Lyme arthritis, and other autoimmune arthritis were combined into one diagnostic group for comparisons—Other Autoimmune Arthritis—because individual groups contained a small number of participants. Furthermore, we performed exploratory analysis to determine if demographics, namely age, sex, or ethnicity, were associated with improvements in the outcome’s variables.

Descriptive statistics were performed to evaluate the demographics of the study population and questions without mutually exclusive answers. Pearson’s chi-squared test was used in the analysis of categorical variables. Numerical Rating Pain Scale point reduction and percent pain reduction were evaluated among arthritis groups with nonparametric Kruskal–Wallis tests. Individual pairwise comparisons were evaluated with Mann–Whitney *U* tests. The Kruskal-Wallis test was used to evaluate trends in pain, physical function, sleep quality (Likert), and pain reduction (NRS) associated with age, sex, arthritis subtype, dose, frequency, and length of CBD use. Multivariate logistic regression models incorporating age, sex, arthritis subtype, dose, frequency, and length of CBD use were used to analyze the effect on other medication reduction or cessation. A two-tailed *p*-value less than 0.05 was considered statistically significant. All statistical analyses were performed with SPSS Statistics V26 (IBM Corporation, Armonk, NY).

## Results

Most participants who completed the survey had tried CBD for symptomatic relief of arthritis (70%). Of the participants who had not tried CBD (*N*=164, 30%), 49.6% needed more information or evidence, 24% were unsure of side effects, 15% had concerns over the legality and drug testing, and 6% stated it was too expensive. Patients that used CBD were younger than patients who had not used CBD, *X*^2^ (*N*=604), *P*<.001, and were more likely from a Western state (22.7% versus 11.9%, respectively) *X*^2^ (*N*=604), *P*=.01. The remaining socioeconomic demographics were not different between the two groups (Table 1).

**Table 1:** Socioeconomic demographics of 604 survey participants with arthritis who use CBD and those who do not use CBD

Demographics	Used CBD (*N*=428) *N* (%)	Not Used CBD (*N*=176) *N* (%)	*P*-value
Age (y)			
18–34	123 (28.7)	45 (25.6)	<.001*
35–54	202 (47.2)	59 (33.5)	
55–64 years old	60 (14)	43 (24.4)	
65 years and older	43 (10)	29 (16.5)	
Gender			
Female	272 (63.6)	121 (68.8)	.14
Male	139 (32.5)	53 (30.1)	
Prefer not to say	17 (4.0)	2 (1.1)	
Ethnicity			
White	357 (83.4)	151 (85.8)	.83
Asian/Pacific Islander	8 (1.8)	3 (1.7)	
African American	17 (4.0)	9 (5.1)	
Hispanic or Latino	18 (4.2)	5 (2.8)	
Native American	5 (1.2)	0 (0)	
Other	16 (3.7)	3 (1.7)	
Not disclosed	7 (1.6)	5 (2.8)	
Geography^a^			
Other country	64 (15.0)	21 (11.9)	.01*
Midwest	68 (15.9)	35 (19.9)	
Northeast	95 (22.2)	52 (29.5)	
South	104 (24.3)	47 (26.7)	
West	97 (22.7)	21 (11.9)	
Education			
High school	96 (22.4)	33 (18.8)	.87
Associate degree	85 (19.9)	38 (21.6)	
Bachelor’s degree	184 (43.0)	77 (43.8)	
Doctorate degree	45 (10.5)	21 (11.9)	
Not disclosed	18 (4.2)	7 (4)	
Income			
<$30,000	102 (23.8)	32 (18.2)	.29
$30,000–$54,999	79 (18.5)	38 (21.6)	
$55,000–$99,999	114 (26.6)	56 (31.8)	
≥100,000	78 (18.2)	25 (14.2)	
Rather not say	55 (12.9)	25 (14.2)	
Type of arthritis			
Osteoarthritis	204 (47.7)	80 (45.5)	.99
Rheumatoid arthritis	142 (33.2)	55 (31.3)	
Other autoimmune arthritis	82 (19.1)	32 (18.2)	
Unsure/missing	0	9 (5.1)	
How did you hear about CBD?^b^			
Friends or family	166 (38.8)		
Internet/social media	151 (35.3)		
Medical personnel	48 (11.2)		
Periodical/television	9 (2.1)		
Unsure	54 (12.6)		
Spoke to the doctor about your CBD use?			
No	195 (45.6)		
Yes	233 (54.4)		
Friends/family use CBD?			
No	75 (17.5)		
Yes	353 (82.5)		

*Y* years, *$* US dollars, *%* percent, *CBD* cannabidiol

Statistical comparisons performed with Pearson chi-squared test between groups

*Significance determined as *P*<0.05

^a^Geographic regions based on US Census Divisions

^b^All patients in the analysis answered “yes” for have you heard of CBD

We compared demographics by type of arthritis and found a significant difference in age (*P*<0.001), with slightly higher proportions of older patients in the OA group compared to RA and other autoimmune arthritis groups. There were no significant differences between groups with regards to gender, ethnicity, location, highest education, or income (Table 2). Participants reported anatomical locations of joint pain, including the knee (*N*=227, 53%), hand (*N*=221, 51.6%), hip (*N*=179, 41.6%), shoulder (*N*=168, 39.3%), wrist (*N*=168, 39.3%), ankle (*N*=112, 26.3%), and elbow (*N*=63, 14.7%). For this question, participants could “check all that apply.” On the basis of the distribution, many patients had pain in multiple joints. Participants reported prior treatment with anti-inflammatories (*N*=392, 91.4%), acetaminophen (*N*=283, 66.0%), physical therapy (*N*=274, 60.7%), intra-articular steroid injection (*N*=197, 45.9%), and opioids (*N*=181, 42.19%).

**Table 2:** Comparison of socioeconomic demographics and CBD utilization based on type of arthritis between group comparisons of participants by type of arthritis and patterns of CBD utilization

	Osteoarthritis	Autoimmune arthritis	Rheumatoid arthritis	
Demographics	*N* (%)	*N* (%)	*N* (%)	*P* value
Age (y)				
18–34	36 (17.6)	25 (30.5)	62 (43.7)	<.001*
35–54	103 (50.5)	36 (43.9)	63 (44.4)	
55–64 years old	40 (19.6)	12 (14.6)	8 (5.6)	
65 years and older	25 (12.3)	9 (11)	9 (6.3)	
Gender				
Female	125 (61.3)	58 (70.7)	89 (62.7)	.319
Male	68 (33.3)	21 (25.6)	50 (35.2)	
Prefer not to say	11 (5.4)	3 (3.7)	3 (2.1)	
Ethnicity				
White	171 (84)	65 (79)	121 (85)	.507
Other ethnicity	33 (16)	17 (21)	21 (15)	
Geography^a^				
Other country	29 (14.2)	14 (17.1)	21 (14.8)	.458
Midwest	28 (13.7)	16 (19.5)	24 (16.9)	
Northeast	45 (22.1)	20 (24.4)	30 (21.1)	
South	60 (29.4)	13 (15.9)	31 (21.8)	
West	42 (20.6)	19 (23.2)	36 (25.4)	
Education				
Associate degree	44 (21.6)	16 (19.5)	25 (17.6)	.314
Bachelor’s degree	80 (39.2)	38 (46.3)	66 (46.5)	
Doctorate degree	23 (11.3)	11 (13.4)	11 (7.7)	
High school	44 (21.6)	16 19.5)	36 (25.4)	
Not disclosed	13 (6.4)	1 (1.2)	4 (2.8)	
Income				
<$30,000	51 (25)	18 (22)	33 (23.2)	.608
$30,000–$54,999	32 (15.7)	18 (22)	29 (20.4)	
$55,000–$99,999	59 (28.9)	16 (19.5)	39 (27.5)	
≥100,000	38 (18.6)	15 (18.3)	25 (17.6)	
Rather not say	24 (11.8)	15 (18.3)	16 (11.3)	

*Y* years, *$* US dollars, *%* percent, *CBD* cannabidiol

Statistical comparisons performed with Pearson chi-squared test between groups

*Significance determined as *P*<0.05

^a^Geographic regions based on US Census Divisions

**Fig. 1:**
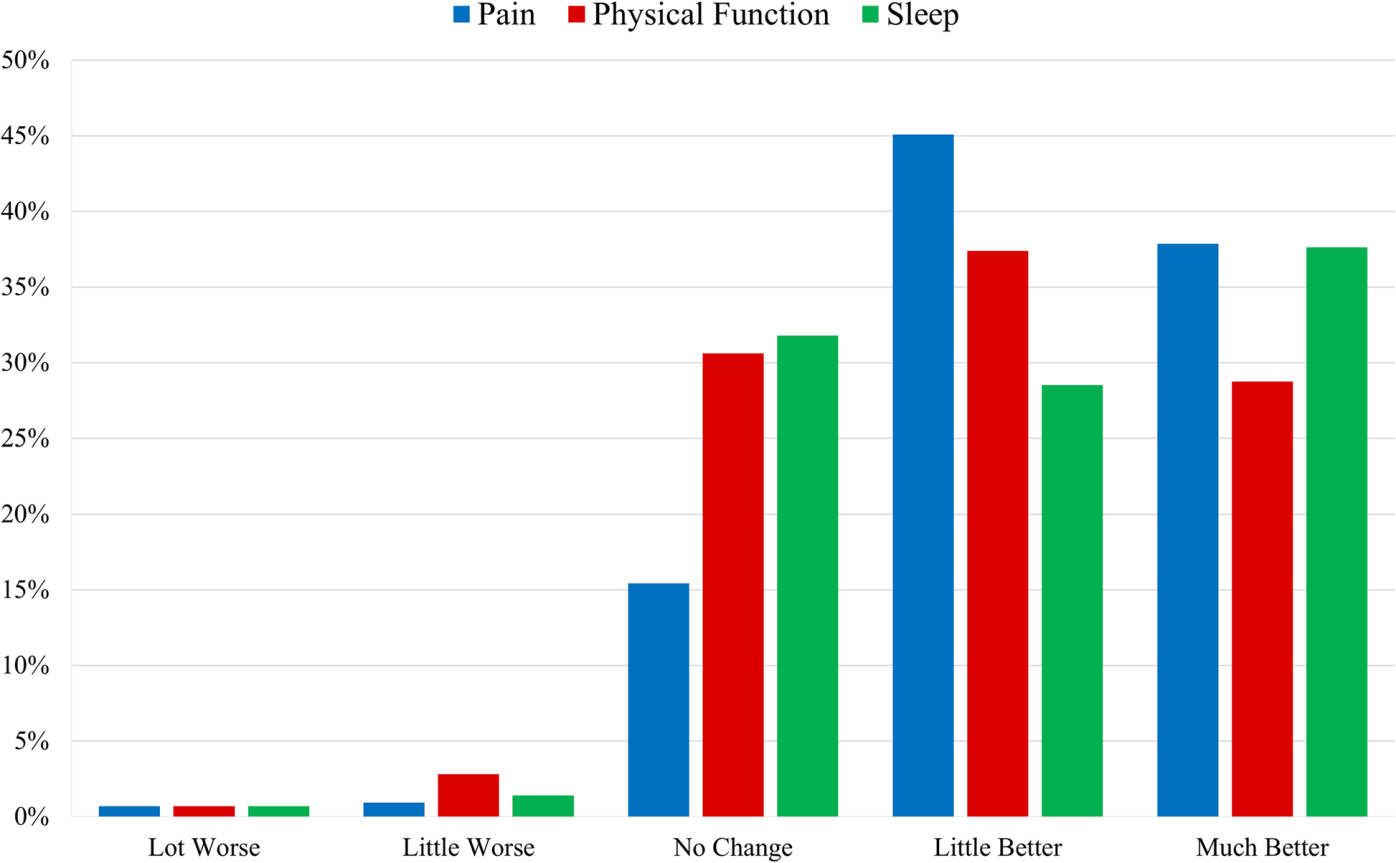
Patients’ global impression of change of pain intensity, physical function, and sleep after using CBD for their joint pain. *“Much better” response has previously been validated as a clinically meaningful effect

For the overall cohort, the majority of participants reported subjective improvements in their symptoms with CBD use. Specifically, 37.9% reported their average daily pain was “much better,” and 45.1% reported their pain was “a little better.” For physical function, 28.7% reported feeling “much better,” and 37.4% reported feeling “a little better.” For sleep quality, 37.6% reported feeling “much better,” and 28.5% reported “a little better.” Lastly, less than 3% of patients reported worsening of their pain, sleep quality, and physical function (Fig. 1).

Analysis by diagnosis (osteoarthritis, RA, or other autoimmune arthritis) was performed. With the exception of age, demographics were not significantly different between groups. Participants with osteoarthritis were more likely to be in the 55–64 age group (*p*=0.001). Differences between groups were seen for subjective improvements in physical function (*p*=0.013) but not for pain (*p*=0.072) or sleep quality (*p*=0.214). Pairwise comparisons between groups for physical function demonstrated that patients with osteoarthritis had more positive responses on the Likert scale than did patients with other autoimmune arthritis (*p*=0.014) (Table 3).

**Table 3:** Patient Global Impression of Change to evaluate perception of cannabidiol’s (CBD) effect on pain intensity, physical function, and sleep based on type of arthritis

After using CBD for your arthritis, how would you best describe its effect on your overall:		Osteoarthritis (*N*=204) Count (%)	Other autoimmune arthritis (*N*=82) Count (%)	Rheumatoid arthritis (*N*=142) Count (%)	*P* value
Pain Intensity?	Lot worse	1 (0.5)	0 (0.0)	2 (1.4)	P=0.072
	Little worse	1 (0.5)	2 (2.4)	1 (0.7)	
	No change	29 (14.2)	18 (22.0)	19 (13.4)	
	Little better	85 (41.7)	36 (43.9)	72 (50.7)	
	Much better	88 (43.1)	26 (31.7)	48 (33.8)	
Physical function?	Lot worse	1 (0.5)	0 (0)	2 (1.4)	P=0.013*
	Little worse	3 (1.5)	3 (3.7)	6 (4.2)	
	No change	55 (27.0)	32 (39.0)	44 (31.0)	
	Little better	77 (37.7)	33 (40.2)	49 (34.5)	
	Much better	68 (33.3)	14 (17.1)	41 (28.9)	
Sleep?	Lot worse	1 (0.5)	0 (0.0)	2 (1.4)	P=0.214
	Little worse	1 (0.5)	2 (2.4)	3 (2.1)	
	No change	65 (31.9)	31 (37.8)	40 (28.2)	
	Little better	58 (28.4)	25 (30.5)	39 (27.5)	
	Much better	79 (38.7)	24 (29.3)	58 (40.8)	

Questions based off Patient Global Impression of Change, 5-point Likert scale

Comparative statistics performed with Kruskal-Wallis based on diagnosis type

*%* percentage

*Significance determined as *P*<0.05

In our assessment of pain reduction after using CBD, participants were asked to rate their pain before and after using a numerical pain score (0–10). The overall cohort reported significant reductions in pain after CBD use, with a 44% reduction in numerical pain score and 2.58-point reduction (*p*<0.001). Comparisons between diagnostic groups can be seen in Table 3. Pairwise comparisons demonstrated that the osteoarthritis group had greater percentage reduction and point reduction compared to RA and other autoimmune arthritis (*p*<0.001) (Table 4).

**Table 4:** Comparison based on diagnosis for question: on a scale of 0–10 with 0 being no pain and 10 being the worst pain imaginable, how would you rate your average daily pain due to your joint condition?

	Total (*N*=428)	Osteoarthritis (*N*=205)	Other autoimmune arthritis (*N*=83)	Rheumatoid arthritis (*N*=143)	
	Mean ± SD	Mean ± SD	Mean ± SD	Mean ± SD	*P*-value
Prior to CBD use?	5.71 ± 1.84	6.2 ± 1.8	5.2 ± 1.9	5.3 ± 1.7	*P*<0.001*
After CBD use?	3.14 ± 1.97	3.1 ± 2.0	3.0 ± 2.0	3.2 ± 1.9	*P*=0.495
Reduction in pain (%)	44 ± 32	48 ± 31	38 ± 37	41 ± 30	*P*=0.020*
Absolute score reduction	2.58 ± 2.07	3.09 ± 2.27	2.13 ± 2.10	2.10 ± 1.51	*P*<0.001*

Comparative statistics performed with Kruskal-Wallis based on diagnosis type

*SD* standard deviation, *%* percentage

*Significance determined as *P*<0.05

In noting differences in frequency (*p*<0.001) and length (*p*<0.001) of CBD use among arthritis groups (Table 5), we performed a trend analysis to further evaluate. There were differences in daily dosage between groups. However, these differences were largely due to a higher proportion of individuals with autoimmune arthritis not knowing their daily dosage (Table 5). Increasing frequency of CBD use was associated with improvements in pain intensity, physical function, and sleep quality (*p*<0.001). Furthermore, this increasing frequency was also associated with greater pain reduction according to the NRS (*p*<0.001). Similarly, increasing length of CBD use was associated with improvements in pain intensity, physical function, sleep quality (*p*<0.001), and greater NRS pain reduction (*p*<0.001).

**Table 5:** Utilization of CBD in 428 patients with arthritis based on type of arthritis

	Osteoarthritis	Autoimmune arthritis	Rheumatoid arthritis	
	*N* (%)	*N* (%)	*N* (%)	*P* value
Frequency				
<Once/month	10 (4.9)	2 (2.4)	7 (4.9)	<.001*
Monthly	6 (2.9)	1 (1.2)	8 (5.6)	
Weekly	21 (10.3)	2 (2.4)	19 (13.4)	
Daily	65 (31.9)	22 (26.8)	47 (33.1)	
>Once/day	82 (40.2)	29 (35.4)	43 (30.3)	
Not specified	20 (9.8)	26 (31.7)	18 (12.7)	
Duration				
<1 month	14 (6.9)	4 (4.9)	19 (13.4)	.002*
1–6 months	52 (25.5)	19 (23.2)	26 (18.3)	
6–12 months	36 (17.6)	15 (18.3)	24 (16.9)	
1–3 years	49 (24)	19 (23.2)	36 (25.4)	
>3 years	34 (16.7)	3 (3.7)	17 (12)	
Not specified	19 (9.3)	22 (26.8)	20 (14.1)	
Daily dose				
≤25 mg	83 (40.7)	30 (36.6)	64 (45.1)	<.001*
26-75 mg	54 (26.5)	21 (25.6)	30 (21.1)	
>75mg	49 (24)	4 (4.9)	27 (19)	
Not specified	18 (8.8)	27 (32.9)	21 (14.8)	

*Mg* milligrams, *%* percentage, < = less than, > = greater than, ≤ = less than or equal to

Statistical comparisons performed with Pearson chi-squared test between groups

*Significance determined as *P*<0.05

### Reduction and/or discontinuation of other medications

Most respondents using CBD for joint pain reported a reduction or cessation of other medications due to CBD use (*N*=259, 60.5%), including a reduction in anti-inflammatories (*N*=129, 31.1%), discontinuation of anti-inflammatories (*N*=76, 17.8%), reduction in acetaminophen (*N*=78, 18.2%), discontinuation of acetaminophen (*N*=76, 17.8%), reduction in opioids (*N*=36, 8.6%), and discontinuation of opioids (*N*=81, 18.9%) (Table 6). Among the different arthritis groups, a higher percentage of patients with osteoarthritis (*N*=135, 66.2%) reduced or discontinued medications, as compared to other autoimmune (*N*=44, 53.7%) and RA (*N*=80, 56.3%) respondents (*p*=0.068). Furthermore, participants who reduced or stopped medications reported increasing frequency of CBD use (*p*<0.001) and overall length of time utilizing CBD (*p*=0.004).

**Table 6:** Number of patients reporting reduced or discontinued use of opioids, anti-inflammatories, and acetaminophen since using CBD

Medication	Reduced use, (%)	Discontinued use, (%)
Opioids	36 (8.4)	81 (18.9)
Anti-inflammatories	129 (30.1)	76 (17.8)
Acetaminophen	78 (18.2)	76 (17.8)

Count and percentage (%) of patients who indicated reducing drug intake and/or discontinuing drug use in each category. Patients were allowed to “check all that apply” resulting in the ability to check that they reduced or discontinued drugs in multiple categories

*%* percentage

Considering these findings, we performed a logistic regression analysis controlling for age and gender to determine the influence of arthritis diagnosis and the frequency and length of CBD use on the reduction/cessation of other medications. Compared to participants with osteoarthritis, participants in the other autoimmune group were 19% more likely (OR=1.19, 95% CI: 0.53–2.66; *p*=0.679) to reduce or stop the use of other medications, and participants in the RA group were 37% less likely (OR=0.63, 95% CI: 0.35–1.12; *p* = 0.116) to reduce or stop the use of other medications (Table 7). For frequency of CBD use, those that used it once a day (OR=5.98, 95% CI: 1.68–21.32; *p*=0.006) and more than once a day (OR=16.90, 95% CI: 4.63–61.72; *p*<0.001) were more likely to reduce or stop other medications as compared to those taking CBD less than once a month (Table 7). Compared to participants with a history of taking CBD for less than 30 days, those that had taken CBD for over three years were 3.61 times more likely to reduce or cease other medications (95% CI: 1.22–10.68; *p*=0.020) (Table 7).

**Table 7:** Logistic regression to analyze the effect of variables for those who selected yes for the question “Did you reduce or discontinue the use of other pharmacologic treatment modalities?”

Variable	OR	95% CI	*P* value
Type of arthritis			
Osteoarthritis	Ref.		
Other autoimmune arthritis	1.19	0.53–2.66	0.679
Rheumatoid arthritis	0.63	0.35–1.12	0.116
Frequency of CBD use			
< Once a month	Ref.		
Once a month	4.15	0.83–20.83	0.084
Once a week	2.46	0.62–9.74	0.199
Once a day	5.98	1.68–21.32	0.006*
More than once a day	16.91	4.63–61.72	<0.001*
Duration of CBD use			
Less than 30 days	Ref.		
1–6 months	1.18	0.50–2.75	0.71
6 months–1 year	2.42	0.96–6.10	0.061
1–3 years	2.38	0.99–5.68	0.052
>3 years	3.61	1.22–10.69	0.020*

Logistical regression model controlled for age, gender, and ethnicity as covariates

*CBD* cannabidiol, *OR* odds ratio, *CI* confidence interval, *%* percent, *Ref.* reference

*Statistically significant (*P* < 0.05)

### Side effects

**Fig. 2:**
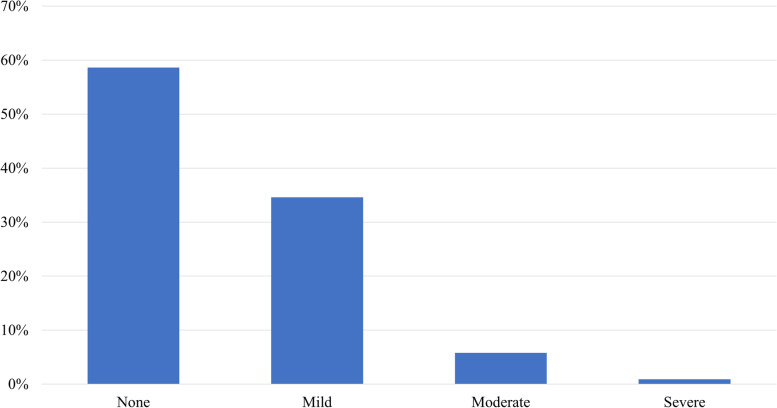
Side effect distribution and severity

**Fig. 3:**
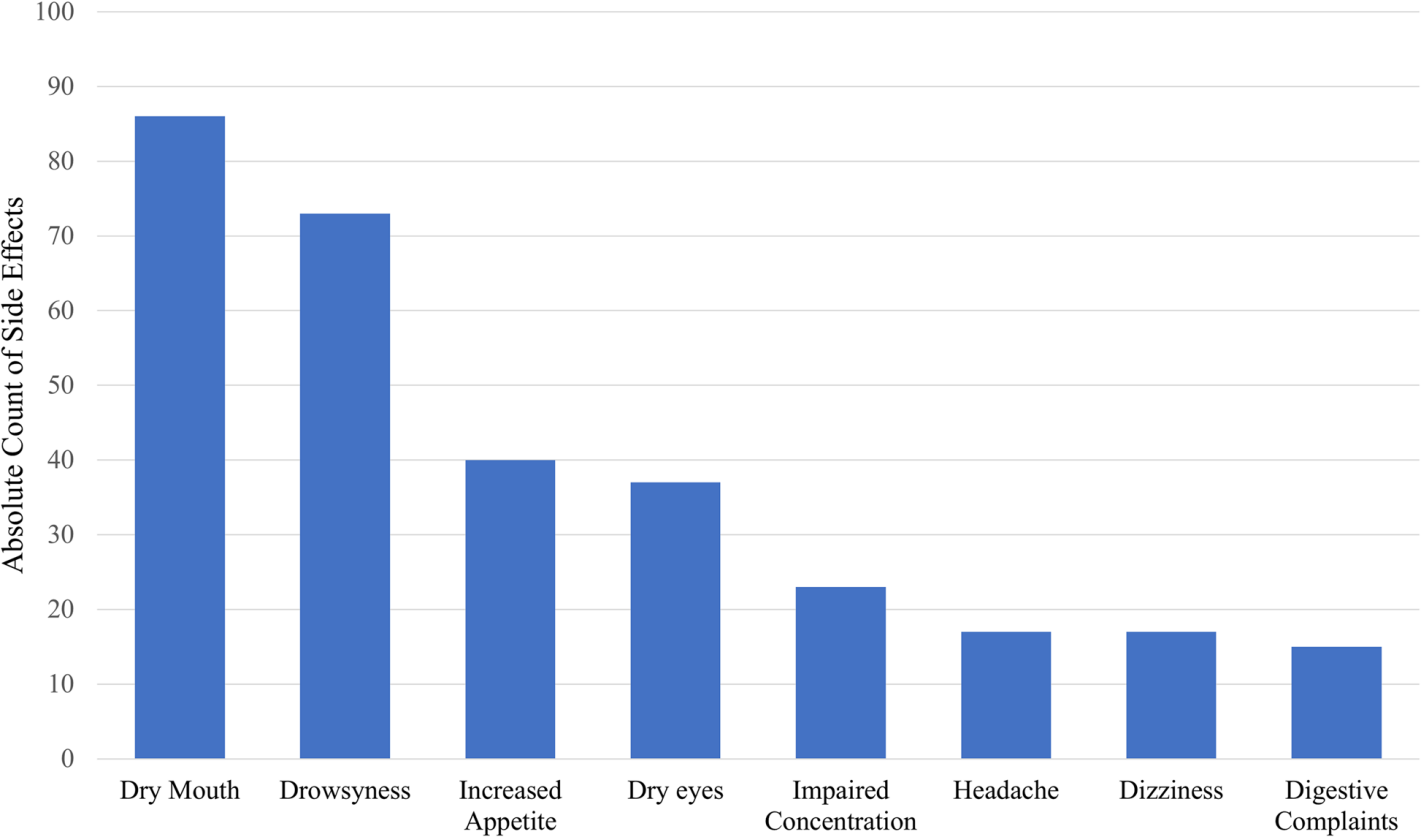
Number of patients reporting the most common side effects

In our study population, 41% of respondents taking CBD for their arthritic pain reported at least one side effect. Of reported side effects, 84% were considered mild, 14% moderate, and 2% severe. The most frequently reported side effects, listed in descending order, were dry mouth (*N*=86, 20%), drowsiness/somnolence (*N*=73, 17%), increased or decreased appetite (*N*=40, 9%), dry eyes (*N*=37, 8.6%), impaired concentration (*N*=23, 5.4%), dizziness (*N*=17, 4%), headache (*N*=17, 4%), and digestive complaints (*N*=15, 3.5%) (Figs. 2 and 3).

## Discussion

The rise in CBD accessibility is in part due to the passage of the Hemp Farming Act of 2018, which removed hemp with less than 0.3% THC from its previous Schedule I controlled substance designation (Comer, 2018). Marketing efforts have largely contributed to the rising popularity of CBD, and it has been touted as a treatment for numerous ailments. Cannabinoids offer an interesting future and relatively novel modality for pain reduction in musculoskeletal disease. The use of CBD for arthritic conditions is beginning to garner attention and may provide a therapeutic avenue with a favorable side effect profile.

In the present study, we found a high prevalence of CBD use to treat joint pain secondary to arthritis. More than half of individuals (60.5%) reported a reduction or discontinuation of opioids, acetaminophen, and/or anti-inflammatories. In terms of the perceived effects of CBD on pain, physical function, and sleep quality, many patients using CBD reported symptomatic improvements. Furthermore, we sought to determine if participants with different types of arthritis had differences in perceived benefits of CBD. Subgroup analysis based on arthritis etiology failed to show differences between groups in terms of pain improvement and sleep quality. The osteoarthritis group had improved subjective physical function scores compared to the autoimmune arthritis group; however, it is unknown if this statistical finding is clinically meaningful. In terms of pain improvement, participants reported a 44% reduction in pain and a 2.58-point difference in the NRS after using CBD. The magnitude of pain alleviation in our study participants, as measured by percentage reduction in pain or point difference, both surpass the clinically important differences reported in literature (2-point reduction or 30% reduction in pain, respectively) (Farrar et al., 2001, Salaffi et al., 2004). Subgroup analysis demonstrated that patients with osteoarthritis reported greater reductions in NRS score than RA and other autoimmune arthritis groups, which may suggest that the efficacy of CBD may be better in patients with osteoarthritis. Research involving CBD for arthritis pain is lacking; however, there have been clinical studies involving CBD and chronic pain. Gulbransen et al. found improvements in quality of life, pain, depression, and sleep quality in 400 patients with non-cancer-related chronic pain. Patients with arthritic conditions were included in their chronic pain cohort, but they did not stratify by reason for chronic pain in their analysis (Gulbransen et al., 2020a, b).

Two of the more commonly utilized medications that participants used for pain relief for arthritis were non-steroidal anti-inflammatory (NSAIDs) and acetaminophen. Boureau et al. compared ibuprofen and paracetamol in patients with arthritis of the knee or hip and found superior pain reduction with NSAID use. Treatment with NSAIDs resulted in a 2.3-point reduction whereas paracetamol resulted in a 1.5-point reduction (Boureau et al., 2004). A systematic review that investigated the use of acetaminophen for pain reduction reported only minimal reductions in pain for patients suffering from hip and knee arthritis, questioning the true efficacy (Leopoldino et al., 2019). Additionally, they found that when comparing acetaminophen to placebo, there was only a 3% absolute reduction in pain and relative reduction in pain of 5% (Leopoldino et al., 2019). The use of topical NSAIDs has also been studied as an alternative to oral NSAIDs, due to the unwanted systemic side effects of the latter. A large meta-analysis found only a minimal pain reduction for topical NSAIDs versus placebo and importantly it was noted that at no time point did topical NSAIDs reach the MCID (Zeng et al., 2018). These conflicting findings support the need for safe, alternative treatments for patients to manage their pain.

One of the particularly interesting findings of this investigation was the proportion of respondents who reduced or discontinued the use of other medications (60.5%). Medications frequently prescribed to manage the symptoms of arthritis can have unwanted side effects. The use of anti-inflammatories has been associated with numerous complications, including the risk of serious gastrointestinal bleeding, ulcerations, and cardiovascular events (Wongrakpanich et al., 2018). Furthermore, it has been reported that up to 50% of patients with arthritis are dissatisfied with the efficacy of anti-inflammatories to treat their pain (Taylor et al., 2013). In our study cohort, of those patients that reduced or discontinued medications after starting CBD, 31.1% reduced anti-inflammatory medications and 17.8% discontinued them altogether.

Despite a lack of evidence supporting long-term opioid use for arthritic pain, a recent study evaluating prescribing practices found that 12.8% of patients were prescribed opioid medications for knee arthritis (Gwam et al., 2021). The U.S. Centers for Disease Control and Prevention (CDC) reported nearly 450,000 opioid-related deaths in the past decade. Given the severity of the current opioid epidemic, finding alternative treatments for pain is vital to public health (Mattson et al., 2021). In our cohort, 8.6% of patients reduced and 18.9% discontinued the use of opioids. These findings suggest that CBD could be an alternative to opioids for the treatment of arthritic pain. Cannabinoids have been shown effective in human trials for chronic pain (Campbell et al., 2018; Moeller-Bertram et al., 2019; Wiese & Wilson-Poe, 2018). In a recent open-label study involving patients with chronic pain and more than 2 years of opioid use, 53.2% of participants were able to reduce opioid medications, lessen their pain, and improve sleep quality after 8 weeks of consuming oral CBD hemp extract (Capano et al., 2020). These findings are congruent with what we observed in our cohort.

Between the different arthritis types, a larger percentage of patients with osteoarthritis (66.2%) reported reductions in other medications used compared to RA (56.3%) and autoimmune arthritis (53.7%). Additionally, we noted that increased dosing frequency and length of use of CBD led to higher rates of other medication reduction and cessation. When controlling for age, dose, gender, the type of arthritis was not found to be an independent predictor. Patients who used CBD more than once a day (OR: 16.9, *p* < 0.001) and once a day (OR: 5.89, *p* = 0.006) were more likely to reduce or stop other medications, when compared to those who took the medication less than once a month. Similarly, patients taking CBD for a period of more than three years were 3.6 times more likely to reduce or cease other medications, when compared to those taking CBD for less than 30 days. One important consideration is that patients who reduce or stop other medications may also be more likely to use CBD regularly and for longer durations as a replacement for the alternative medications.

Despite all of the study participants having a diagnosis that was made by a physician, only 45.6% had spoken to a health care professional about CBD use for their condition. This may be because CBD is readily accessible and does not require a prescription. Another possibility is that healthcare professionals may not be discussing CBD use with their patients. Although CBD is available for purchase without consultation with a doctor, the authors recommend that patients discuss this with their doctor before use. Cannabidiol could have unwanted side effects and/or interactions with other medications. Most of the literature regarding the adverse events of CBD use has come from clinical trials for the FDA-approved indication to treat seizure disorders (Corroon & Kight, 2018). Recently, the World Health Organization released a critical review on CBD and found it is generally well tolerated with a good safety profile and does not have abuse potential (Expert Committee on Drug Dependence, 2018). Previous cross-sectional analyses have found at least one reported side effect in 29–59% of patients using CBD for different indications which is in concordance with our findings (41%) (Corroon & Phillips, 2018; Devitt, 2019; Moltke & Hindocha, 2021). Furthermore, most of the reported side effects in our study were mild (84%), which echoes the findings in clinical trials and cross-sectional studies.

The study has limitations that must be considered when interpreting these results. First, the survey used was created by the authors and was based on clinical expertise as well as other published studies (Corroon and Phillips, 2018, Bhamra et al. 2021, Moltke et al, Schilling et al. 2021, Moeller-Bertram et al. 2019, Devitt 2019). Due to this novel survey design, there is concern for the validity of the survey. In order to improve validity and accuracy, the survey underwent multiple rounds of internal review to reduce errors and to monitor the quality of questions as described by Passmore et al. 2002. Importantly, the structure of the survey was based on the Likert scale and NRS, which have been validated in the literature and therefore improve the validity of the custom survey used in the investigation (Safikhani et al. 2018, Passmore et al. 2002). Second, the study population was a self-selected sampling of convenience and hence might not be representative of the general population. Of the respondents, 176 (30%) patients with arthritis had not tried CBD, whereas 428 (70%) endorsed CBD use. Two recent studies found a prevalence of CBD use in patients with arthritis to be between 24 and 57% which is lower than our findings (Deckey et al., 2021, Nowell, 2019). The authors feel it is unlikely that 70% of patients with arthritis in the general population have tried CBD. This higher prevalence may be due to a selection bias secondary to patients who have used CBD being more likely to participate in the study. Third, patients with polarizing views on experiences using cannabinoids—whether they are positive or negative—may be more likely to have participated in the study, which could lead to either positive or negative bias. Additionally, because the survey was distributed online through social media posting and patient representative groups, patients with arthritis who have limited connectivity to the internet are potentially underrepresented. Fourth, patients submitted subjective reports of their symptoms, and the survey was not distributed in a controlled manner immediately after CBD use, making the results susceptible to recall bias. Designing a prospective study may allow assessment of pain before and after CBD use in real time to circumvent recall bias limitation. Lastly, a large proportion of patients did not know the type of CBD product (e.g., isolate, broad spectrum, full spectrum) they consumed, and therefore, the authors were unable to evaluate associations between patient-perceived effects of CBD and product type. Different types of CBD and the various modes of administration could have possible varying effects, which we were not able to capture in this investigation.

## Conclusion

Clinicians and patients should be aware of the various alternative therapeutic options available to treat their symptoms of arthritis, especially in light of the increased accessibility to cannabidiol products. The present study, while exploratory in nature, suggests there may be therapeutic benefits to CBD use and highlights the need for research in a field where the science lags behind popular use. Future research should focus on exploring the benefits of CBD use in this patient population with well-controlled clinical trials.

## Supplementary Information

**Additional file 1.**

**Additional file 2.**
