# Supplementary material for: Cannabidiol as a treatment for arthritis and joint pain: an exploratory cross-sectional study
Source: J Cannabis Res. 2022 Aug 24;4:47. doi: 10.1186/s42238-022-00154-9 (PMC9400326; doi:10.1186/s42238-022-00154-9)
Supplement: Supplementary file 1 — Additional file 1. [file 42238_2022_154_MOESM1_ESM.pdf]

CBD & Arthritis Survey

This survey is designed to evaluate the incidence of CBD use in individuals with arthritis. All survey responses are collected anonymously. Please answer all questions below.

Patient Demographics

How old are you?

- ◯ 18 years old
- ◯ 18-24 years old
- ◯ 25-34 years old
- ◯ 35-44 years old
- ◯ 45-54 years old
- ◯ 55-64 years old
- ◯ 65-74 years old
- ◯ 75 years and older
- ◯ Prefer not to say

What is your gender?

- ◯ Male
- ◯ Female
- ◯ Prefer not to say

What is your ethnicity?

- ◯ White
- ◯ Hispanic or Latino
- ◯ Black or African American
- ◯ Asian/Pacific Islander
- ◯ Native American or American Indian
- ◯ Other
- ◯ Not disclosed

What state do you live in?

◯ Alabama
◯ Alaska
◯ Arizona
◯ Arkansas
◯ California
◯ Colorado
◯ Connecticut
◯ Delaware
◯ Florida
◯ Georgia
◯ Hawaii
◯ Idaho
◯ Illinois
◯ Indiana
◯ Iowa
◯ Kansas
◯ Kentucky
◯ Louisiana
◯ Maine
◯ Maryland
◯ Massachusetts
◯ Michigan
◯ Minnesota
◯ Mississippi
◯ Missouri
◯ Montana
◯ Nebraska
◯ Nevada
◯ New Hampshire
◯ New Jersey
◯ New Mexico
◯ New York
◯ North Carolina
◯ North Dakota
◯ Ohio
◯ Oklahoma
◯ Oregon
◯ Pennsylvania
◯ Rhode Island
◯ South Carolina
◯ South Dakota
◯ Tennessee
◯ Texas
◯ Utah
◯ Vermont
◯ Virginia
◯ Washington
◯ West Virginia
◯ Wisconsin
◯ Wyoming
◯ Other (Not United States)

IF Other, Please Specify what country

____

What is your highest level of education?

- ◯ No schooling completed
- ◯ Nursery school to 8th grade
- ◯ High school graduate or equivalent
- ◯ Associate degree
- ◯ Bachelor's degree
- ◯ Doctorate degree
- ◯ Not disclosed

What is your occupation?

- ◯ Arts, Design, Entertainment, Sports, and Media Occupations
- ◯ Legal Occupations
- ◯ Life, Physical, and Social Science Occupations
- ◯ Management Occupations
- ◯ Construction and Extraction Occupations
- ◯ Healthcare Practitioners and Technical Occupations
- ◯ Computer and Mathematical Occupations
- ◯ Office and Administrative Support Occupations
- ◯ Healthcare Support Occupations
- ◯ Production Occupations
- ◯ Food Preparation and Serving Related Occupations
- ◯ Architecture and Engineering Occupations
- ◯ Installation, Maintenance, and Repair Occupations
- ◯ Building and Grounds Cleaning and Maintenance Occupations
- ◯ Sales and Related Occupations
- ◯ Community and Social Service Occupations
- ◯ Personal Care and Service Occupations
- ◯ Farming, Fishing, and Forestry Occupations
- ◯ Protective Service Occupations
- ◯ Education, Training, and Library Occupations
- ◯ Business and Financial Operations Occupations
- ◯ Transportation and Materials Moving Occupations
- ◯ Other (please specify)

Occupation (Other)

____

What is your average yearly income?

- ◯ < $30,000
- ◯ $31,000-$55,000
- ◯ $56,000-$75,000
- ◯ $76,000-$99,000
- ◯ $100,000-$150,000
- ◯ >$150,000
- ◯ I would rather not comment

Perception/Use of Cannabidiol (CBD)

Have you ever heard of cannabadiols (CBD)?	◯ Yes<br>◯ No<br>◯ Unsure
How did you heard about CBD?	◯ Internet<br>◯ Television<br>◯ Periodical<br>◯ Social Media<br>◯ Friends or family<br>◯ Medical personnel<br>◯ Unsure
Do you have a form of arthritis and/or an inflammatory joint condition that has been diagnosed by your medical doctor or physician?	☐ Yes<br>☐ No

Your Joint Pain

Disclaimer: If you answered no to the previous question, and have no history of joint pain or arthritis conditions your survey should have ended.

What type of arthritis and/or inflammatory joint condition do you have? (Please select all that apply)

- ☐ Osteoarthritis
- ☐ Rheumatoid arthritis
- ☐ Psoriatic arthritis
- ☐ Other autoimmune diagnosis (Lupus, Lyme, etc.)
- ☐ Unsure

Does your joint arthritis cause you pain?

- ◯ Yes
- ◯ No

On a scale of 0-10 with 0 being no pain and 10 being the worst pain imaginable, how would you rate your average daily pain due to your joint condition?

- ◯ 0
- ◯ 1
- ◯ 2
- ◯ 3
- ◯ 4
- ◯ 5
- ◯ 6
- ◯ 7
- ◯ 8
- ◯ 9
- ◯ 10

In reference to your joint pain, what joint causes you the most discomfort/pain? (Please select all that apply)

- ☐ Knee
- ☐ Hip
- ☐ Shoulder
- ☐ Elbow
- ☐ Ankle
- ☐ Wrist
- ☐ Hand

What treatments have you tried for your joint pain? (Please select all that apply)

- ☐ Physical therapy
- ☐ Steroid Injection
- ☐ Viscosupplementation (gel shot)
- ☐ PRP (platelet rich plasma)
- ☐ Supplements
- ☐ Acupuncture
- ☐ Opioids
- ☐ Tylenol
- ☐ Anti-inflammatories (Advil, Ibuprofen, Naproxen, Meloxcicam, etc.)
- ☐ CBD
- ☐ Other
- ☐ Unsure

Have you ever spoken to your doctor about using Cannabidiol (CBD)?

- ◯ Yes
- ◯ No

Do you have family and/or friends that use CBD?

- ◯ Yes
- ◯ No

Have you ever tried Cannabidiol (CBD) for your joint pain?

- ◯ Yes
- ◯ No

If you haven't tried CBD, is there a reason why? (Please select all that apply)	☐ Unsure about side effects<br>☐ Need more information<br>☐ Afraid it is illegal<br>☐ do not have access to CBD<br>☐ Other (please specify)
Other (please specify)	____________________
Would you be open to learning more about CBD to help manage your joint pain?	◯ Yes<br>◯ No<br>◯ Unsure

Your Experience Using CBD

What method of CBD delivery have you tried?

☐ Smoking or Vaporizing
☐ Ingesting in food or beverage
☐ Ingesting in pill or capsule
☐ Sublingually (tincture, drops, spray)
☐ Topically (cream, ointment, balm, lotion)

What type of CBD do you use?

☐ CBD from hemp (little to no THC)
☐ CBD from or with Cannabis (some THC)
☐ Both A and B
☐ Not sure

What doses (mg) of CBD do you use daily?

☐ ≤25
☐ 26mg-75mg
☐ >75mg

How often do you use CBD for your joint pain?

◯ More than once a day
◯ Once a day
◯ Once a week
◯ Once a month
◯ Less than once a month
◯ Not specified

How long have you been using CBD for your joint pain?

◯ Less than 30 days
◯ Between 1 and 6 months
◯ 6 Months to a year
◯ 1 to 3 years
◯ Over 3 years
◯ Not specified

Prior to using CBD to treat your joint pain how would you rate your pain on a scale to 0-10, with 0 being no pain and 10 being the worst pain imaginable?

◯ 0
◯ 1
◯ 2
◯ 3
◯ 4
◯ 5
◯ 6
◯ 7
◯ 8
◯ 9
◯ 10

After using CBD to treat your joint pain how would you rate your pain on a scale to 0-10, with 0 being no pain and 10 being the worst pain imaginable?

◯ 0
◯ 1
◯ 2
◯ 3
◯ 4
◯ 5
◯ 6
◯ 7
◯ 8
◯ 9
◯ 10

After using CBD how does this affect your joint pain?

◯ Lot worse
◯ Little worse
◯ No change
◯ Little better
◯ Much better

After using CBD how does this affect your pain intensity?

◯ Lot worse
◯ Little worse
◯ No change
◯ Little better
◯ Much better

After using CBD how does this affect your physical function?

◯ Lot worse
◯ Little worse
◯ No change
◯ Little better
◯ Much better

After using CBD how does this affect your sleep?

◯ Lot worse
◯ Little worse
◯ No change
◯ Little better
◯ Much better

How satisfied are you with using CBD to alleviate your joint pain?

◯ Very Satisfied
◯ Satisfied
◯ Neutral
◯ Dissatisfied
◯ Very Dissatisfied

Has using CBD for joint pain reduced the use of other medications?

◯ No
◯ Yes
◯ Unsure

What medications have been reduced or stopped from CBD use?

☐ Less opioids
☐ stopped opioids
☐ less Anti-inflammatories
☐ stopped Anti-inflammatories
☐ less Tylenol
☐ stopped Tylenol
☐ None
☐ Unsure

What are side effects have you have had from using CBD? Select all that apply.

☐ Dry mouth
☐ Tiredness
☐ Bloodshot or dry eye
☐ Increased appetite
☐ Headache
☐ Dizziness
☐ Digestive upset
☐ Increased pulse/heart rate
☐ Impaired concentration
☐ Increased sensitivity
☐ Anxiety
☐ Distorted perception
☐ Impaired coordination
☐ Other
☐ No side effects

Of the side effects experienced how would you rate the severity?

◯ Mild
◯ Moderate
◯ Severe
◯ No side effects

Do you think there is good clinical evidence that CBD is helpful?	◯ Yes<br>◯ No<br>◯ Unsure
How much would you be willing to pay for CBD products in order to treat your arthritis pain?	◯ < $25<br>◯ $26-$50<br>◯ $51-$75<br>◯ $76-$100<br>◯ $101-$125<br>◯ $126-$150<br>◯ >$150
Based on your experience with CBD for joint pain, would you recommend to others?	◯ Yes<br>◯ No<br>◯ Unsure
