# Supplementary material for: Cannabidiol as a treatment for arthritis and joint pain: an exploratory cross-sectional study
Source: J Cannabis Res. 2022 Aug 24;4:47. doi: 10.1186/s42238-022-00154-9 (PMC9400326; doi:10.1186/s42238-022-00154-9)
Supplement: Supplementary file 2 — Additional file 2. [file 42238_2022_154_MOESM2_ESM.docx]

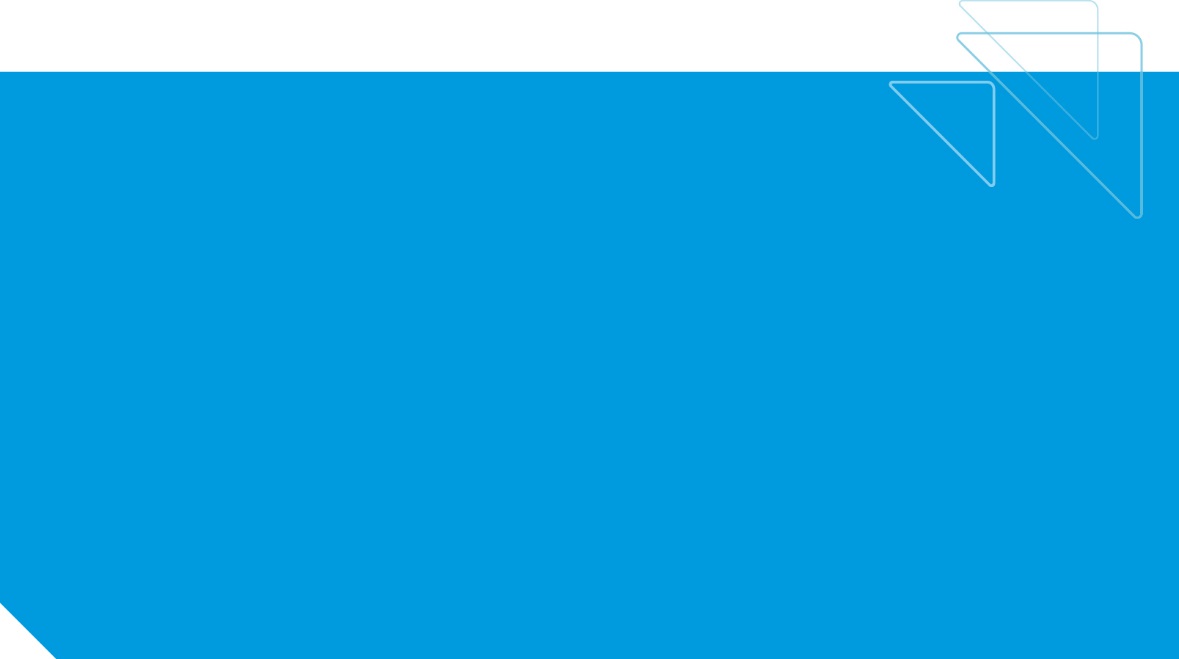
**Anonymous Survey on Cannabidiol (CBD) Use Perception and Prevalence in Individuals with Joint Pain**

**Background:**

- Arthritis is one of the most common ailments of the musculoskeletal system
- Pre-clinical studies in arthritic animal models have shown that CBD reduces inflammation and pain behaviors
- Positive public perception and interest has led to an increase in the utilization of CBD for various medical conditions

**Purpose of Study:**

- Our study hopes to determine if patients with various forms of arthritis and joint pain are using CBD, as well as their perceived impact on its utility for their symptoms

**Participant Involvement:**

- You must be over 18 years old to complete the survey
- Participants should have some form of arthritis that causes them joint pain
- Your information is valuable even if you **Have Not** tried CBD before, CBD use is not a prerequisite
- Participation involves completing an online survey, which should take about 6-7 minutes to complete
- The survey will be **anonymous**
- We ask that you try to answer all questions. However, if there are any questions that you would prefer to skip, simply leave the answer blank

**Works Cited ?:**

1. Hammell, D. C., Zhang, L. P., Ma, F., Abshire, S. M., McIlwrath, S. L., Stinchcomb, A. L., & Westlund, K. N. (2016). Transdermal cannabidiol reduces inflammation and pain-related behaviours in a rat model of arthritis. *European Journal of Pain (United Kingdom)*, *20*(6), 936–948. https://doi.org/10.1002/ejp.818

**Ways to Access Survey:**

**Direct Link:** [**https://redcap.northwell.edu/surveys/?s=WJWE8DEKXM**](https://redcap.northwell.edu/surveys/?s=WJWE8DEKXM)

**Go To:**

<https://redcap.northwell.edu/surveys/>

Enter code: HNJKPXYX9

**QR Code with personal smart phone:**

**
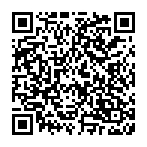
**

| **Northwell Health Orthopaedic Institute**  [OrthoResearch@northwell.edu](mailto:OrthoResearch@northwell.edu) |
| --- |
|  |
